# Estimating the value of point-of-care HPV testing in three low- and middle-income countries: a modeling study

**DOI:** 10.1186/s12885-017-3786-3

**Published:** 2017-11-25

**Authors:** Nicole G. Campos, Vivien Tsu, Jose Jeronimo, Mercy Mvundura, Jane J. Kim

**Affiliations:** 1 Center for Health Decision Science, Department of Health Policy and Management, Harvard T.H. Chan School of Public Health, 718 Huntington Avenue, 2nd Floor, Boston, Massachusetts 02115 USA; 2 PATH, Reproductive Health Global Program, P.O. Box 900922, Seattle, Washington, USA; 3 Global Coalition against Cervical Cancer, Arlington, Virginia, USA; 4 PATH, Devices and Tools Program, P.O. Box 90922, Seattle, Washington, USA

**Keywords:** Cancer screening, Cost-effectiveness analysis, Human papillomavirus (HPV), HPV DNA tests, Uterine cervical neoplasms, Decision analysis

## Abstract

**Background:**

Where resources are available, the World Health Organization recommends cervical cancer screening with human papillomavirus (HPV) DNA testing and subsequent treatment of HPV-positive women with timely cryotherapy. Newer technologies may facilitate a same-day screen-and-treat approach, but these testing systems are generally too expensive for widespread use in low-resource settings.

**Methods:**

To assess the value of a hypothetical point-of-care HPV test, we used a mathematical simulation model of the natural history of HPV and data from the START-UP multi-site demonstration project to estimate the health benefits and costs associated with a shift from a 2-visit approach (requiring a return visit for treatment) to 1-visit HPV testing (i.e., screen-and-treat). We estimated the incremental net monetary benefit (INMB), which represents the maximum additional lifetime cost per woman that could be incurred for a new point-of-care HPV test to be cost-effective, depending on expected loss to follow-up between visits (LTFU) in a given setting.

**Results:**

For screening three times in a lifetime at 100% coverage of the target population, when LTFU was 10%, the INMB of the 1-visit relative to the 2-visit approach was I$13 in India, I$36 in Nicaragua, and I$17 in Uganda. If LTFU was 30% or greater, the INMB values for the 1-visit approach in all countries was equivalent to or exceeded total lifetime costs associated with screening three times in a lifetime. At a LTFU level of 70%, the INMB of the 1-visit approach was I$127 in India, I$399 in Nicaragua, and I$121 in Uganda.

**Conclusions:**

These findings indicate that point-of-care technology for cervical cancer screening may be worthy of high investment if linkage to treatment can be assured, particularly in settings where LTFU is high.

**Electronic supplementary material:**

The online version of this article (10.1186/s12885-017-3786-3) contains supplementary material, which is available to authorized users.

## Background

Cervical cancer is preventable through screening and treatment of precancerous lesions. Accordingly, 85% of the global burden resides in the developing world, where access to health care is lacking [1]. The knowledge that cervical cancer is caused by persistent infection with one or more oncogenic human papillomavirus (HPV) types [2] has led to advances in screening technology, including HPV DNA tests that are highly sensitive to detect precancer and cancer [3]. In addition to being clinically validated, one round of HPV DNA testing in women over age 30 reduced advanced cervical cancer incidence and mortality by 50% in a large randomized trial in India [4], demonstrating the potential for population-level health gains with a single round of screening. Where resources are available, the World Health Organization (WHO) thus recommends a “screen-and-treat” strategy for women aged 30 to 49 years, screening with HPV testing and treating eligible HPV-positive women with timely cryotherapy [5]. Where resources for organized screening with HPV testing are limited, the WHO recommends visual inspection with acetic acid (VIA), which can provide immediate results at a low cost, but is considerably less sensitive and necessitates stringent quality control measures and provider training.

An organized screening program relying on HPV DNA testing requires health infrastructure that can accommodate laboratory processing and the need for at least two visits in a screening episode [6]. While the *care*HPV test— a validated low-cost HPV test developed through a multinational collaborative public-private partnership— is suitable for use in low-resource settings due to its minimal laboratory requirements [7], laboratory processing time is approximately 4 hours. Furthermore, the careHPV testing system is designed to be run in batch mode [8], with optimal use at 90 samples per batch; few clinics in low-resource settings can achieve this high screening volume in a single day. These processing time and batch size constraints hinder same-day results and treatment for HPV-positive women. Given the substantial barriers to returning to the health facility in many low-resource settings [9–11], the need for at least two visits (first, for administration of the screening test and second, for receiving results and treatment if screen-positive) signifies that many women in need of treatment might never receive it.

Newer technologies and next-generation HPV tests address some of the limitations associated with existing tests by reducing lab processing time to approximately 1 hour per sample and running in a non-batch mode, which may facilitate a same-day screen-and-treat approach with a validated HPV test [8, 12, 42]. Still, these testing systems are generally too expensive for widespread use in low- and middle-income countries. To estimate the value of a hypothetical HPV test that reduces the number of required visits for a screening episode, we used a mathematical simulation model of the natural history of HPV and cervical cancer, as well as cost and test performance data from the START-UP multi-site demonstration project, to project the change in health benefits, costs, and net monetary benefits of a single-visit approach in India, Nicaragua, and Uganda.

## Methods

### Analytic overview

We used an existing individual-based Monte Carlo simulation model of the natural history of HPV and cervical cancer to estimate lifetime health and economic outcomes associated with screening with HPV DNA testing. We considered 2- and 1-visit screen-and-treat strategies (with treatment provided immediately following receipt of a positive result) in order to estimate the value of reducing the number of required visits per screening episode. The model was calibrated separately to epidemiologic data from India, Nicaragua, and Uganda [13]. Test performance and cost data were drawn from the START-UP multi-site demonstration project conducted in India (Hyderabad), Nicaragua (Masaya Province), and Uganda (Kampala); a fourth site in India was not included in this evaluation [14, 15]. Model-projected outcomes included health benefits— in terms of reductions in lifetime risk for cervical cancer incidence and gains in life expectancy— and lifetime costs (in 2011 international dollars [I$]). Consistent with guidelines for cost-effectiveness analysis [16–18], we adopted a societal perspective, including costs irrespective of the payer, and discounted future costs and life-years at a rate of 3% per year to account for time preferences.

**Fig. 1:**
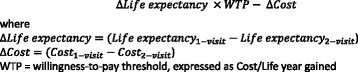
Equation for calculation of the incremental net monetary benefit (INMB). Values for life expectancy and the average lifetime cost per woman (the expected value of costs associated with screening, management of screen-positive women, and cancer treatment) were model outcomes. While there is no universal criterion that defines a threshold cost-effectiveness ratio for societal WTP (in terms of cost per life-year gained), we considered the heuristic that an intervention with an ICER less than the country’s gross domestic product (GDP) per capita would be “very cost-effective” [19] and less than three times GDP per capita would be “cost-effective”

Cost-effectiveness analysis relies on the incremental cost-effectiveness ratio (ICER), defined as the incremental cost of a strategy divided by its incremental benefit, compared with the next most costly strategy. The ICER is an indicator of an intervention’s efficiency; when an intervention has an ICER that is less than the willingness to pay (WTP) for a health unit (i.e., life years gained), it may be considered “good value for money.” While there is no universal criterion that defines a threshold cost-effectiveness ratio for societal WTP, we considered the heuristic that an intervention with an ICER less than the country’s gross domestic product (GDP) per capita would be “very cost-effective” [19] and less than three times GDP per capita would be “cost-effective”. To measure the added value of shifting from a 2-visit screening approach (i.e., with loss-to-follow-up) to a 1-visit screening approach with immediate treatment (i.e., no loss to follow-up), we calculated the incremental net monetary benefit (INMB) for the 1-visit strategy relative to the 2-visit approach. The INMB translates the incremental health benefit (additional life-years gained from shifting to a 1-visit strategy) into monetary terms for a given WTP threshold (by multiplying the life years gained by the WTP) and then subtracts the incremental cost (the change in the expected lifetime cost per woman from shifting to a 1-visit strategy) [20] (Fig. 1). Thus, the INMB is the maximum dollar amount per woman by which the cost of an intervention can be increased to achieve an improvement while remaining “cost-effective” (i.e., having an ICER below the WTP threshold).

### Mathematical simulation model

The natural history model of cervical carcinogenesis comprises mutually exclusive health states, including type-specific HPV infection status, grade of precancer (i.e., cervical intraepithelial neoplasia [CIN] grade 2 or 3), and stage of invasive cancer [13, 21]. Individual girls enter the model at age 9 years with a healthy cervix and transition between health states on a monthly basis until death; transition probabilities may vary by age, HPV type, duration of infection or precancerous lesion status, and prior HPV infection. Cancer detection can occur through symptoms or via screening. Death from background mortality can occur from any health state, and excess stage-dependent mortality can occur from cervical cancer after its onset. The model tracks disease progression and regression, clinical events, and economic outcomes over the lifetime for each individual woman; individual outcomes are then aggregated for analysis.

The model parameterization process, including calibration, has been previously described [13, 21–23]. Briefly, we established baseline parameter values for the natural history component of the model using longitudinal data [24–28]. To reflect heterogeneity in age- and type-specific HPV incidence between settings, as well as natural immunity following initial infection and uncertainty in progression and regression of precancer, we set plausible ranges around these input parameter values. Repeated model simulations in the absence of any intervention selected a single random value from the plausible range for each uncertain parameter, creating a unique natural history input parameter set. We then computed a goodness-of-fit score by summing the log-likelihood of model-projected outcomes for each unique parameter set to represent the quality of fit to country-specific epidemiologic data (i.e., calibration targets). For each country, we selected the top 50 input parameter sets that produced good fit to the epidemiologic data to use in analyses as a form of probabilistic sensitivity analysis [21, 23, 29]. Model fit to empirical data on age-specific high-risk HPV prevalence data from the START-UP projects and age-specific cancer incidence is displayed in Additional file 1. We report results as the mean and range of outcomes across these top 50 parameter sets (data available in Additional file 2).

### Screening strategies

We assumed screening with HPV DNA testing took place three times in a woman’s lifetime at ages 30, 35, & 40 years, as these screening ages and interval have been demonstrated in a previous study to have ICERs less than GDP per capita in each setting [13]. To determine the maximum dollar amount that the cost of screening can be increased to reduce the number of clinic visits per screening episode while maintaining cost-effectiveness, we assumed population screening coverage was 100%. We varied loss to follow-up (LTFU) between each health facility visit from 10% to 70%, in increments of 10%. We considered two strategies at each level of LTFU, including 2-visit screen-and-treat at the clinic (i.e., the best case given existing technology) and 1-visit screen-and-treat at the clinic. The pathway of care for the 2-visit strategy involved screening by a healthcare provider with the *care*HPV test at an initial clinic visit; women who were not LTFU returned for a second visit to obtain results. If they were HPV-positive and eligible, women would receive same-day cryotherapy at the results visit. In the 1-visit strategy, women were assumed to receive screening by a healthcare provider with a hypothetical HPV test (with test performance equivalent to *care*HPV administered by a provider) at the clinic and, if HPV-positive and eligible, same-day cryotherapy. Because we assumed comparable test performance for the 2-visit and 1-visit strategies rather than potentially reduced test sensitivity for the 1-visit strategy, the INMB represents the maximum cost increase that could be tolerated for a reduced number of visits, while maintaining an ICER below the WTP threshold. Treatment protocols for women who were not eligible for immediate cryotherapy, and management following treatment, were based on current practice in each country and are documented in Additional file 1. Test performance and treatment parameters are presented in Table 1 [14, 15, 22, 30–35].

**Table 1:** Baseline values for model variables^a^

Variable [Reference]	India	Nicaragua	Uganda
Population coverage of screening program	100%	100%	100%
Loss to follow-up per visit^b^	10-70%	10-70%	10-70%
Proportion of eligible women receiving immediate cryotherapy following positive *care*HPV result^b^	100%	100%	100%
*care*HPV (cervical specimen) sensitivity/specificity for CIN2+ [15]	90% / 95%	78% / 89%	89% / 82%
Test sensitivity/specificity for CIN1+, colposcopy^c^	50% / 96%	95% / 68%	95% / 51%
Eligibility for cryotherapy [22]			
No lesion or CIN1	100%	100%	100%
CIN2	85%	85%	85%
CIN3	75%	75%	75%
Cancer	10%	10%	10%
Effectiveness of cryotherapy [22, 30–32]	92%	92%	92%
Effectiveness of cryotherapy/LEEP following colposcopy [22, 31]	96%	96%	96%
Direct medical costs by procedure [14, 15]^d^			
*care*HPV (cervical specimen)^e^	9.24	15.61	8.78
Colposcopy^f^	9.86	15.25	7.08
Colposcopy and biopsy^f^	30.06	39.48	32.90
Cryotherapy	38.13	33.04	13.49
LEEP	NA	133.64	139.54
Cytology (follow-up post-treatment)^g^	15.15	13.71	12.25
Direct non-medical costs^d^			
Transportation (round-trip, clinic) [22, 33, 34]	0.08	0.69	4.46
Transportation (round-trip, secondary facility) [22, 33, 34]	15.29	2.75	10.87
Women’s time (per hour) [35]	1.14	1.41	0.68
Treatment of local cancer (FIGO stages 1a-2a)[22, 33, 34]^d,h^	1821	3322	888
Treatment of regional/distant cancer (FIGO stages ≥2b)[22, 33, 34]^d,h^	2652	4268	1176

^a^CIN: cervical intraepithelial neoplasia; FIGO: International Federation of Gynecology and Obstetrics; LEEP: loop electrosurgical excision procedure. Further details on unit cost assumptions are available in Additional file 1

^b^Loss to follow-up is defined as the proportion of women who do not return for each subsequent clinical encounter, relative to the previous visit. For the 2-visit screen-and-treat strategy, this applied to the results/cryotherapy visit, as well as subsequent visits for diagnostic confirmation and treatment among women who are ineligible for cryotherapy in a screen-and-treat approach. For the 1-visit screen-and-treat strategy, loss to follow-up only applied to diagnostic confirmation and treatment visits among women who are ineligible for immediate cryotherapy. All women who received a positive *care*HPV result and presented to the clinic and were deemed eligible were assumed to receive immediate cryotherapy

^c^Test performance characteristics of colposcopy in the START-UP demonstration projects were derived from the worst diagnosis of the local pathologist relative to the worst diagnosis by a quality control pathologist (gold standard); we applied the treatment threshold of CIN1+, although this was not the treatment threshold in START-UP. To derive test performance of colposcopy, we excluded histological classifications that were inadequate or with a histological classification other than negative, CIN1, CIN2, CIN3, or cancer. Because CIN1 is not a true underlying health state in the model, performance of colposcopy in the model is based on the underlying health states of no lesion, HPV infection, CIN2, or CIN3. For a treatment threshold of CIN1, we weighted sensitivity of colposcopy for women with HPV based on the country-specific prevalence of CIN1 among women with HPV infections in the START-UP studies

^d^All costs are in 2011 international dollars (I$). The location of service delivery for each procedure, as well as time spent traveling, waiting for, and receiving care by procedure and country, are presented in Additional file 1. In the START-UP study, procedures were performed at secondary or tertiary facilities, and costs may over- or under-estimate costs at primary health facilities due to differences in volume of procedures and overhead costs

^e^This includes the cost of the careHPV test, which was assumed to be I$5

^f^The proportion of colposcopies that were accompanied by a biopsy was drawn from START-UP data as follows: 93.1% (India); 95.6% (Uganda); and 99.5% (Nicaragua), in the absence of data from actual practice in low-resource settings

^g^Protocols for follow-up after treatment varied by country, and are described in Additional file 1

^h^All cancer costs presented include the value of women’s time spent pursuing care and transportation to health facilities

### Cost data

Cost data have been published elsewhere [13] but are summarized in Table 1. Direct medical costs of screening, diagnosis, and treatment of precancerous lesions were drawn from the START-UP study sites, and included staff time, clinical supplies, drugs, clinical equipment, laboratory staff time, laboratory supplies, and laboratory equipment. We converted local currency units to 2011 I$, a hypothetical currency that provides a means of translating and comparing costs among countries, taking into account differences in purchasing power. We assumed the *care*HPV test kit was a tradable good valued at US$5.

Transportation costs and the cost of women’s time spent traveling, waiting for, and receiving care were dependent upon the facility level and were derived from START-UP data and the published literature, as previously described [14, 15, 22, 33, 34]. Costs associated with cancer care by stage included direct medical costs, women’s time costs, and transportation costs.

The costs for the 1-visit screen-and-treat strategy differed from the 2-visit strategy because women’s travel and waiting time, as well as transportation costs, for the second visit were removed; both scenarios included the provider and woman’s time spent on actual results delivery, as well as additional waiting time for the cryotherapy procedure if screen-positive. Direct medical costs for screening with HPV testing (and treatment procedures) were the same for both strategies, as we assumed that the direct medical costs associated with new technologies allowing for 1-visit screening would be at least as costly as provider-collection of *care*HPV samples. Because the unknown incremental costs of hypothetical new technologies are not included in the analysis, the INMB represents the total expected cost per woman (including both direct medical and non-medical components) that could be incurred over and above the existing direct medical costs of screening with provider-collection of *care*HPV samples, in order for a new HPV test (associated with a single visit) to be cost-effective [36].

## Results

**Fig. 2:**
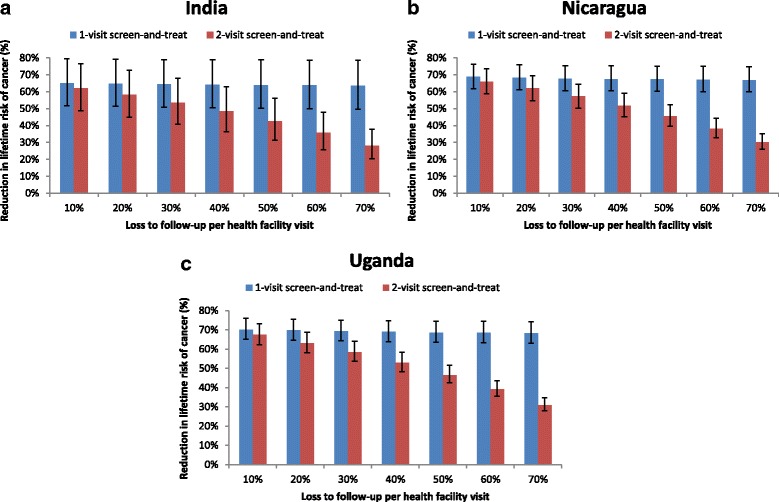
Reduction in lifetime risk of cancer associated with the 2-visit versus the 1-visit screen-and-treat strategy. Reduction in lifetime risk of cancer (y-axis) is displayed for screening three times in a lifetime at ages 30, 35, and 40 years with HPV DNA testing, as loss to follow-up per health facility visit is varied from 10% to 70% (x-axis) in **a**) India; **b**) Nicaragua; and **c**) Uganda. Cancer reduction associated with the 2-visit screen-and-treat strategy is represented by the red bars; the 1-visit screen-and-treat is represented by the blue bars. Error bars display the range in cancer reduction across the 50 calibrated input parameter sets

Figure 2 shows the reduction in cancer incidence associated with the 2-visit and 1-visit screen-and-treat strategies in each country, as LTFU per health facility visit is varied from 10% to 70%. Screening three times in a lifetime at 30, 35, and 40 years with a 2-visit screen-and-treat program available to all eligible women and a LTFU rate of 10% reduces cancer incidence by 62.0% in India, 66.0% in Nicaragua, and 67.4% in Uganda. Estimates for the 1-visit strategy were similar to the 2-visit strategy when LTFU was 10%: 65% in India, 68.8% in Nicaragua, and 70.1% in Uganda. However, as LTFU increased, reduction in cancer risk associated with the 1-visit strategy remained stable in each country, while the health impact of the 2-visit approach diminished substantially. At 40% LTFU, the 1-visit strategy reduced cancer risk by 64% in India, 67.5% in Nicaragua, and 68.9% in Uganda; by comparison, the 2-visit strategy reduced cancer risk by 48.4% in India, 51.8% in Nicaragua, and 52.8% in Uganda. When LTFU reached 70%, the 1-visit strategy reduced cancer risk by 63.5% in India, 66.9% in Nicaragua, and 68.3% in Uganda, while the 2-visit strategy reduced cancer risk by 28.2%, 30.2%, and 30.9% in India, Nicaragua, and Uganda, respectively.

Table 2 presents the discounted lifetime costs, life expectancy, and INMB assuming a WTP threshold equivalent to each country’s GDP per capita. In India and Uganda, the 2-visit scenario was associated with higher costs than the 1-visit scenario until LTFU reached 50% in India and 70% in Uganda. These higher costs were attributable to the higher time and transportation costs for women to attend the clinic twice, as opposed to only once. At high levels of LTFU, the costs associated with the 2-visit approach declined as fewer women received treatment, while the 1-visit scenario costs remained stable. In Nicaragua, the cost of the 2-visit scenario increased steadily with LTFU— as fewer high-risk women received treatment, the high cost of cancer treatment in this setting offset the lower costs resulting from reduced treatment, women’s time, and transportation.

**Table 2:** Health outcomes, costs, and incremental net monetary benefits of shifting from a 2-visit to a 1-visit approach for cervical cancer screening and treatment of precancer^a^

	India (GDP per capita: I$5450)				Nicaragua (GDP per capita: I$4690)				Uganda (GDP per capita: I$1690)			
Strategy^b^	Cancer incidence reduction, %^c^	Lifetime cost (2011 I$)^d^	Life expectancy^e^	INMB (1× GDP per capita threshold)^f^	Cancer incidence reduction, %^c^	Lifetime cost (2011 I$)^d^	Life expectancy^e^	INMB (1× GDP per capita threshold)^f^	Cancer incidence reduction, %^c^	Lifetime cost (2011 I$)^d^	Life expectancy^e^	INMB (1× GDP per capita threshold)^f^
No screening	–	8.87 (7.05-12.54)	27.78539 (27.76486-27.79362)	–	–	42.67 (37.28-50.31)	28.58210 (28.54475-28.61306)	–	–	12.42 (10.96-14.16)	25.20221 (25.17432-25.22855)	–
LTFU: 10%												
2-visit	62.0 (48.8-76.6)	29.68 (27.25-32.02)	27.82880 (27.82371-27.83431)	–	66.0 (58.9-73.6)	53.03 (48.86-56.89)	28.73581 (28.72337-28.74977)	–	67.4 (62.3-73.1)	45.66 (43.01-47.78)	25.33856 (25.33166-25.34855)	–
1-visit	65.0 (51.8-79.4)	27.32 (24.81-29.72)	27.83081 (27.82611-27.83594)	13 (11-16)	68.8 (61.7-76.3)	48.69 (44.60-52.56)	28.74246 (28.73123-28.75554)	36 (29-42)	70.1 (65.0-75.9)	37.75 (34.87-40.12)	25.34406 (25.33770-25.35278)	17 (15-19)
LTFU: 20%												
2-visit	58.1 (45.1-72.8)	28.92 (26.55-31.22)	27.82567 (27.82006-27.83160)	–	61.9 (54.8-69.5)	53.51 (49.16-57.58)	28.72533 (28.71125-28.74107)	–	63.2 (58.1-68.8)	43.91 (41.45-45.79)	25.32940 (25.32187-25.34090)	–
1-visit	64.7 (51.4-79.1)	27.31 (24.79-29.72)	27.83013 (27.82534-27.83545)	26 (21-32)	68.2 (61.1-75.8)	48.82 (44.72-52.74)	28.74042 (28.72857-28.75389)	75 (61-88)	69.6 (64.6-75.4)	37.70 (34.81-40.07)	25.34217 (25.33572-25.35127)	28 (24-31)
LTFU: 30%												
2-visit	53.5 (40.9-68.0)	28.21 (25.91-30.45)	27.82200 (27.81527-27.82855)	–	57.2 (50.2-64.5)	54.25 (49.76-58.61)	28.71253 (28.69672-28.73052)	–	58.4 (53.6-64.0)	42.21 (39.94-43.82)	25.31800 (25.30857-25.33155)	–
1-visit	64.3 (50.9-78.9)	27.31 (24.77-29.73)	27.82940 (27.82447-27.83494)	41 (35-54)	67.8 (60.7-75.4)	48.93 (44.80-52.87)	28.73814 (28.72585-28.75192)	125 (104-148)	69.2 (64.2-75.1)	37.65 (34.76-40.02)	25.34011 (25.33346-25.34958)	42 (35-48)
LTFU: 40%												
2-visit	48.4 (36.2-62.9)	27.54 (25.26-29.73)	27.78180 (27.81012-27.82500)	–	51.8 (45.3-59.0)	55.24 (50.64-59.90)	28.69851 (28.68045-28.71844)	–	52.8 (48.3-58.3)	40.61 (38.52-41.95)	25.30545 (25.29383-25.32110)	–
1-visit	64.0 (50.4-78.9)	27.30 (24.74-29.73)	27.82872 (27.82352-27.83453)	59 (51-77)	67.5 (60.5-75.2)	48.99 (44.80-52.92)	28.73577 (28.72275-28.75003)	181 (153-213)	68.9 (63.8-74.8)	37.60 (34.71-39.97)	25.33789 (25.33107-25.34820)	58 (49-66)
LTFU: 50%												
2-visit	42.6 (31.3-56.2)	26.92 (24.71-29.22)	27.81361 (27.80433-27.82065)	–	45.6 (39.5-52.2)	56.62 (51.93-61.68)	28.68319 (28.66223-28.70486)	–	46.4 (42.4-51.5)	39.08 (27.19-40.42)	25.29166 (25.27809-25.30957)	–
1-visit	63.9 (50.2-78.9)	27.29 (24.70-29.70)	27.82818 (27.82289-27.83407)	79 (69-105)	67.2 (60.2-75.0)	49.01 (44.81-52.95)	28.73386 (28.72017-28.74855)	245 (211-289)	68.6 (63.5-74.5)	37.54 (34.66-39.91)	25.33606 (25.32892-25.34684)	77 (65-88)
LTFU: 60%												
2-visit	35.7 (25.7-47.8)	26.39 (24.31-28.90)	27.80868 (27.79781-27.81552)	–	38.3 (32.9-44.2)	58.38 (53.62-63.84)	28.66565 (28.65149-28.68919)	–	39.1 (35.4-43.6)	37.66 (35.86-39.03)	25.27631 (25.26027-25.29619)	–
1-visit	63.7 (49.9-78.7)	27.28 (24.69-29.71)	27.82763 (27.82222-27.83379)	102 (88-137)	67.0 (60.0-74.9)	49.04 (44.83-52.96)	28.73182 (28.71765-28.74704)	320 (279-377)	68.4 (63.2-74.3)	37.49 (34.60-39.87)	25.33429 (25.32690-25.34550)	98 (84-113)
LTFU: 70%												
2-visit	28.2 (20.3-37.9)	25.89 (23.96-28.62)	27.80362 (27.79067-27.81054)	–	30.2 (26.0-35.2)	60.44 (55.49-66.41)	28.64712 (28.62014-28.67254)	–	30.9 (27.9-34.7)	36.33 (34.63-37.76)	25.26014 (25.32487-25.34428)	–
1-visit	63.5 (49.6-78.7)	27.27 (24.68-29.69)	27.82722 (27.82182-27.83347)	127 (106-174)	66.9 (59.9-74.8)	49.04 (44.82-52.97)	28.72980 (28.71518-28.74542)	399 (350-468)	68.3 (63.1-74.2)	37.44 (34.55-39.82)	25.33251 (25.32487-25.34428)	121 (105-140)

^a^Average values represent the outcomes using 50 calibrated parameter sets for each country; parentheses indicate the minimum and maximum values across 50 calibrated parameter sets. GDP: gross domestic product; I$: international dollars; INMB: incremental net monetary benefit; LTFU: loss to follow-up

^b^Strategies are listed in order of increasing health benefit, and include either 2-visit screen-and-treat or 1-visit screen-and-treat for women aged 30, 35, and 40 years. Under both strategies, screening coverage was 100%, with LTFU for each clinical encounter. For the 2-visit strategy, LTFU applied to the results/treatment visit; for both strategies, LTFU applied to diagnostic testing with colposcopy and treatment of colposcopically confirmed CIN1+ for women who were not eligible for immediate cryotherapy at a primary facility

^c^Cancer incidence reduction reflects the percent reduction in lifetime risk of cancer incidence compared to no screening

^d^Total discounted lifetime cost per woman

^e^Total discounted life expectancy

^f^The INMB for 1-visit screen-and-treat is calculated against 2-visit screen-and-treat, at the specified level of LTFU. The INMB for 1-visit screen-and-treat provides a measure of how much economic investment could be made per woman to achieve a reduction in visits (relative to the 2-visit screen-and-treat scenario) without exceeding a country’s willingness to pay (WTP), at each level of LTFU. We considered each country’s WTP to be equivalent to GDP per capita

Life expectancy associated with the 2-visit strategy was always lower than for the 1-visit strategy, particularly at high levels of LTFU, in accordance with higher cancer risk due to imperfect compliance.

**Fig. 3:**
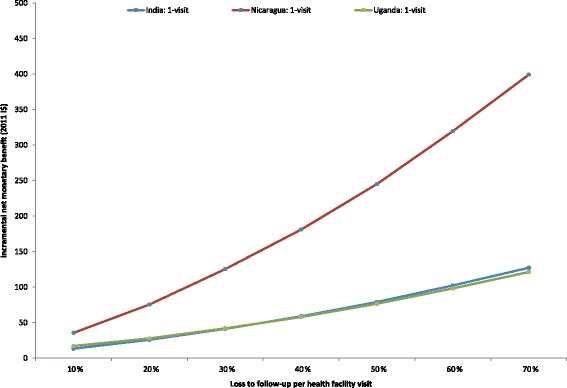
Incremental net monetary benefit of shifting from the 2-visit strategy to the 1-visit strategy. Incremental net monetary benefit (INMB) (in 2011 international dollars, y-axis) is displayed for each country as loss to follow-up (LTFU) per health facility visit is varied from 10% to 70% (x-axis), assuming a willingness-to-pay threshold equivalent to each country’s GDP per capita. For the 2-visit strategy, LTFU applied to the results/cryotherapy visit, as well as subsequent visits for diagnostic confirmation and treatment among women who were ineligible for cryotherapy in a screen-and-treat approach. For the 1-visit screen-and-treat strategy, LTFU only applied to diagnostic confirmation and treatment visits among women who were ineligible for immediate cryotherapy. The INMB for 1-visit HPV testing (relative to 2-visit HPV testing) for India is represented by the blue line, Nicaragua by the red line, and Uganda by the green line

At higher levels of LTFU, shifting from a 2-visit to a 1-visit strategy was associated with higher INMB values (Fig. 3), assuming a WTP threshold equivalent to GDP per capita. This finding indicates that as LTFU increases, reducing the number of clinic visits may be worthy of high investments. INMB values were similar between India and Uganda at all levels of LTFU, although the range of uncertainty was wider in India due to greater variation in cancer incidence across the 50 calibrated input parameter sets (Table 2). INMB values were highest in Nicaragua, where the cost of cancer treatment was high relative to the costs of screening and precancer treatment. When LTFU was 10%, the INMB of the 1-visit relative to the 2-visit scenario was I$13 in India, I$36 in Nicaragua, and I$17 in Uganda. If LTFU was 30% or greater, the INMB values for the 1-visit scenarios in all countries was equivalent to or exceeded total lifetime costs associated with screening three times in a lifetime. When LTFU was 40%, the INMB of the 1-visit scenario was I$59 in India, I$181 in Nicaragua, and I$58 in Uganda. At a LTFU level of 70%, the INMB of the 1-visit scenario was I$127 in India, I$399 in Nicaragua, and I$121 in Uganda.

For a WTP threshold of three times GDP per capita, results are presented in Additional file 1. Findings suggest similar increases in INMB values as LTFU increases, although INMB values are consistently higher at all LTFU levels due to the higher WTP threshold. When LTFU was 10%, the INMB for a 1-visit scenario was I$35 in India, I$98 in Nicaragua, and I$36 in Uganda. When LTFU was 70%, the INMB was I$384 in India, I$1175 in Nicaragua, and I$366 in Uganda.

## Discussion

To assess the value of a hypothetical HPV test that reduces the number of required visits for a screening episode, we estimated the INMB associated with a shift from 2-visit to 1-visit HPV DNA testing. The differences in cost and health impact between these strategies were owing to 1) varying levels of LTFU between health facility visits and 2) women’s time and transportation for required visits. The INMB values can be interpreted as the maximum additional lifetime cost per woman that could be incurred for a new HPV test to be cost-effective, depending on the expected LTFU in a given setting. We found that, for screening three times in a lifetime with 2-visit HPV DNA testing, LTFU for screen-positive women had a profound impact on reduction in cancer risk, with potential health benefits halved as LTFU rose from 10% to 70%. The INMB for the 1-visit screening strategy depended upon LTFU. At low levels of LTFU (e.g., 10%), the INMB values associated with a shift from 2-visit to 1-visit testing were approximately 40% or more of total lifetime costs associated with the 2-visit strategy; at high levels of LTFU (e.g., 70%), the INMB values for shifting from a 2-visit to a 1-visit approach were highest. These findings indicate that where LTFU is particularly high, point-of-care technology may be worthy of high investment if linkage to treatment can be assured for screen-positive women. INMB values were similar in India and Uganda, but much higher in Nicaragua as a result of the high costs of cancer treatment in this setting.

Because we assumed that the cost and performance of a new test would be similar to *care*HPV with provider-collection of cervical samples, the INMB of improving the screening process by shifting from 2-visit to 1-visit screen-and-treat represents the maximum *additional* cost (over and above the direct medical cost of provider-administration of *care*HPV) that could be incurred on average per woman over her lifetime without exceeding the WTP threshold. Thus, the additional costs associated with a hypothetical point-of-care test would likely need to be applied at each of the three screening episodes over a woman’s lifetime, without exceeding the estimated total present value INMB. We used I$ to facilitate comparisons across settings, but we note that for tradable goods, such as potential new technologies, 1 international dollar is equal to 1 U.S. dollar. However, it is likely that interventions to reduce the number of clinic visits will not rely solely on investments in new technology, but also investments in infrastructural improvements and human resources to facilitate access to screening and maximize availability of same-day treatment. In the absence of a point-of-care test, resources could alternatively be spent on interventions to reduce LTFU instead of reducing the number of clinic visits. However, the INMB values for this analysis reflect the reduced costs for women’s time and transportation associated with fewer clinic visits, and the societal value would likely be lower for an intervention that did not also eliminate these costs. It is important to note that the INMB values do not represent a per-woman price threshold for new technologies, nor do they reflect necessary investments required for development of novel technologies; rather, the INMB represents an estimate of total societal resources that could be expended per woman without exceeding the WTP threshold.

GDP per capita is a benchmark for cost-effectiveness promoted by the WHO-CHOICE program [37], and the implied assumption is that a country is willing to pay up to that threshold (or three times that threshold) for a unit of health benefit (e.g., year of life saved). However, supporting data from revealed and stated preference approaches to elicit societal willingness to pay for a year of life saved are often lacking [38]. The use of the GDP per capita threshold for determination of cost-effectiveness may not lead to the best allocation of scarce resources if there are other necessary and feasible interventions with greater value for public health dollars that remain unfunded [38]. Furthermore, information on the value for money is not equivalent to affordability, or the financial impact of a program on a payer’s budget. As Marseilles and colleagues [38] point out, there is no evidence that society will contribute the necessary sums to implement all interventions that meet the WHO’s criteria for cost-effectiveness. We acknowledge that our selection of GDP per capita as a threshold for willingness to pay is somewhat arbitrary, albeit based upon current methodological convention. To more accurately assess the value of health interventions to improve the screening process, better data on willingness to pay for health improvements in low- and middle-income countries are needed.

There are several limitations to this study. We do not present ICERs for the scenarios considered, as the focus of our analysis was to estimate the value of reducing the number of screening visits, considering a baseline of screening three times in a woman’s lifetime with HPV testing. Thus, we do not consider alternative screening tests, frequencies, or ages, which we have considered elsewhere [13], but focus on LTFU as the parameter of interest. The INMB estimates for the 1-visit strategy were generated under assumptions of full screening coverage and perfect compliance to cryotherapy among women who receive positive results. In principle, these estimates represent the maximum economic cost that society would be willing to pay to move from a 2-visit screen-and-treat approach to a 1-visit screen-and-treat approach. In reality, universal coverage and cryotherapy for all eligible women are not realistic, and different technologies or interventions to reduce the number of clinic visits may have different effectiveness in practice, leading to potentially lower return on investments. For instance, the development of a point-of-care screening test may facilitate same-day results, but if cryotherapy is not consistently available onsite, a 1-day screen-and-treat scenario may not be feasible. Specific program costs, including the cost of new technologies, human resources, and infrastructure, will need to be carefully assessed to determine the feasibility of particular improvements in a low-resource setting, and the actual return on investments. In future analyses, we plan to apply the analytic framework presented here to estimate the INMB of other potential improvements in the screening process.

There are limitations surrounding our costing data. While direct medical costs were drawn from the START-UP demonstration projects, we extrapolated average women’s time and transportation costs from other analyses [22, 33], and these likely do not capture the broad geographic variation within a country. Women’s time and transportation costs are a substantial component of total screening costs in low-resource settings [33], and are intimately associated with baseline LTFU rates [10]. If we have underestimated women’s costs, our INMB values may underestimate the societal value of reducing the number of visits per screening episode. We also extrapolated cancer treatment costs from other analyses [22, 33, 34], and our findings of very high INMB values in Nicaragua reflect the high costs of cancer treatment in this setting, as extrapolated from El Salvador. In the absence of data from resource-intensive implementation, geospatial, and time and motion studies, we believe our costing assumptions are reasonable estimates.

Providing cervical cancer screening and treatment of precancer in low-resource settings with poor access to health care is logistically difficult. Major obstacles include women’s time and transportation costs to accessing the clinic, laboratory processing that precludes same-day results, and unreliable access to cryotherapy. Our objective was to estimate the societal value of point-of-care HPV tests. Despite uncertainty in cost data, our findings indicate that economic investment in purchasing and administering such a screening test could be high and still provide good value for public health dollars. The recent unveiling of the GeneXpert Omni (Cepheid, Sunnyvale, California), a portable rapid multi-analyte diagnostic test, may be a promising technologic development to facilitate rapid results for HPV testing [39]. While the Xpert HPV assay can provide results with approximately 1 h of laboratory processing and has been clinically validated against other HPV tests and in a low-resource setting [40, 41], further study is needed to determine whether new point-of-care technologies can improve health outcomes in a real-world setting [40]. If a point-of-care HPV test does not achieve greater rates of patient notification of results and ultimately higher compliance with timely ablative treatment relative to existing technology, the benefits are unlikely to outweigh the costs. Implementation studies are needed to identify the real-world costs associated with wastage, equipment maintenance and working life, quality control measures, and personnel time required for test administration [42], as well as real-world compliance with potential 1-visit approaches.

## Conclusions

There are nearly 1 billion women aged 30 to 49 years [43], who are past the primary target age for HPV vaccination; most of these women have not been screened for cervical cancer. Our model projections suggest that economic investment in a point-of-care HPV test and supporting infrastructure in three low- and middle-income countries could be high and still provide good value for public health dollars. In the near future it will be critical to assess whether new tests entering the market can achieve improved health outcomes in a cost-effective and sustainable manner.

## Additional files

Additional file 1: Supplementary model documentation, methods, and results. This file includes detailed descriptions of the model calibration process, cost data, and supplementary results on the incremental net monetary benefit considering an alternative willingness-to-pay threshold. (DOCX 113 kb)

Additional file 2: Supplementary file_raw data. Raw data (model outputs) used for calculations in the published manuscript. (XLSX 104 kb)

## Abbreviations

CIN: Cervical intraepithelial neoplasia
GDP: Gross domestic product
HPV: Human papillomavirus
I$: International dollars
ICER: Incremental cost-effectiveness ratio
INMB: Incremental net monetary benefit
LTFU: Loss to follow-up
VIA: Visual inspection with acetic acid
WHO: World Health Organization
WTP: Willingness to pay
