# Supplementary material for: Estimating the value of point-of-care HPV testing in three low- and middle-income countries: a modeling study
Source: BMC Cancer. 2017 Nov 25;17:791. doi: 10.1186/s12885-017-3786-3 (PMC5702206; doi:10.1186/s12885-017-3786-3)
Supplement: Supplementary file 1 — Supplementary model documentation, methods, and results. This file includes detailed descriptions of the model calibration process, cost data, and supplementary results on the incremental net monetary benefit considering an alternative willingness-to-pay threshold. (DOCX 113 kb) [file 12885_2017_3786_MOESM1_ESM.docx]

**Supplementary model documentation, methods, and results:**

**Estimating the value of point-of-care HPV testing in three low- and middle-income countries**

This appendix provides additional details on methods, assumptions, and results presented in the main manuscript. A detailed description of the natural history model and parameterization for the United States population has been previously published [[1](#_ENREF_1)]. The adaptation of this model to the populations of India, Nicaragua, and Uganda has been previously described in a separate publication and accompanying appendix [[2](#_ENREF_2)]. We summarize the calibration and parameterization of these models below.

**MODEL CALIBRATION**

**Overview of the calibration process**

Details of the model development process, including initial parameterization and calibration, have been previously published [[1](#_ENREF_1)]. Derivation of model parameter values requires an iterative process involving comprehensive literature reviews, data synthesis and analysis, consultations with experts, and explorations of the influence of uncertain parameters and assumptions in the model. Baseline HPV incidence rates, as a function of genotype and age, were derived from published data from a prospective cohort of sexually active women aged 15-85 years in Bogota, Colombia [[3](#_ENREF_3)]. Because HPV incidence is known to vary by population as a function of sexual behaviors, age-specific HPV incidence and natural immunity following initial infection were considered important candidates for calibration. Transitions occurring from the HPV state (i.e., time-dependent rates of HPV clearance and progression by genotype) were informed by primary longitudinal data from the control arm of the Costa Rica Vaccine Trial [[4](#_ENREF_4)]. Type-specific data on CIN2 and CIN3 regression and progression are limited [[5-10](#_ENREF_5)], so these parameters were also candidates for calibration. Because of the computational intensity of microsimulation models, we selected parameters for calibration based on the availability of 1) a range of plausible values and 2) good empirical data to inform calibration targets (i.e., high-risk HPV prevalence to calibrate HPV incidence rates; cancer incidence to calibrate CIN2 and CIN3 regression and progression rates).

To calibrate the model, we set plausible search ranges around baseline input values for age- and type-specific HPV incidence, as well as natural immunity following initial infection and progression and regression of CIN, and performed repeated model simulations in the absence of any preventive intervention. For each simulation, we randomly selected a single value for each of the uncertain parameters from the identified plausible range, creating a unique vector of parameter values (i.e., parameter “set”). Following over 1,475,000 repeated samplings, we identified the parameter sets with the highest correspondence to the empirical calibration target data by calculating and aggregating the log-likelihood of model-projected outcomes. We used the 50 parameter sets with the highest likelihood scores (i.e., best overall fit to the empirical data) from each country for analysis to capture uncertainty in the model parameters as a form of probabilistic sensitivity analysis. We report results as a mean and a range of outcomes across these top 50 parameter sets; incremental cost-effectiveness ratios are reported as the ratio of the mean costs divided by the mean effects of one strategy versus another across sets.

**Calibration targets**

We assessed model fit by observing projected model outcomes of age-specific prevalence of high-risk HPV and age-specific cancer incidence relative to empirical data. The scoring algorithms for India and Nicaragua included age-specific prevalence of high-risk HPV and age-specific cervical cancer incidence. For Uganda, only age-specific cancer incidence was included in the scoring algorithm, as we observed a better fit to cancer incidence data when we did not include HPV prevalence in the scoring algorithm; however, we still considered visual fit to HPV prevalence to arrive at the final scoring algorithm.

Age-specific prevalence of high-risk HPV was drawn from START-UP data on *care*HPV positivity using a cut-off ratio cut-point of 0.5 relative light units (**Tables A.1, A.2, A.3**). For each age group, we derived a 95% binomial confidence interval around the point prevalence, which comprised the calibration target. The likelihood function for each age group was assumed to follow a binomial distribution.

Age-specific cancer incidence was drawn from registries in *Cancer in Five Continents* [[11](#_ENREF_11)] for India and Uganda, and from Globocan for Nicaragua due to the lack of a cancer registry [[12](#_ENREF_12)] (**Tables A.4, A.5, A.6**). The likelihood function for each age group was assumed to follow a normal distribution.

Composite goodness-of-fit scores for each input parameter set were generated by summing the log likelihood of each model outcome (i.e., age-specific HPV prevalence, age-specific cancer incidence). The 50 input parameter sets with the highest goodness-of-fit scores thus yielded the model outputs that were simultaneously closest to all calibration targets, and were selected for analysis. **Figures A.1, A.2, A.3, A.4, A.5,** and **A.6** display model fit to epidemiologic data on age-specific prevalence of high-risk HPV and age-specific cancer incidence in each country.

**COST DATA**

**Direct Medical Costs: Screening, Diagnosis, and Treatment of Precancerous Lesions**

The direct medical costs of screening, diagnosis, and treatment of precancerous lesions were drawn from the Screening Technologies to Advance Rapid Testing for Cervical Cancer Prevention–Utility and Program Planning (START-UP) demonstration studies in India (Hyderabad), Nicaragua (Masaya Province), and Uganda (Kampala). Direct medical costs included clinical staff time, clinical supplies, drugs, clinical equipment, laboratory staff time, laboratory supplies, and laboratory equipment.

We report costs in 2011 international dollars (I$) to facilitate comparisons across regions. The relevant GDP deflators were applied to local currency units to inflate to year 2011 levels, and local currency units were then converted to international dollars by means of purchasing power parity (PPP) exchange rates [[13](#_ENREF_13)]. The exceptions were for equipment, which was generally procured in the United States, and the cost of the careHPV test kit, which was assumed to be US$5. For these tradable goods, one international dollar is equivalent to one U.S. dollar. Costs are reported in **Table 1** of the main manuscript.

**Cost of Cancer Care by Stage**

Costs associated with cancer care by stage (Local vs. Regional or Distant), including direct medical costs, women's time costs for time spent receiving care, women's transportation costs to health facilities, and cancer staging costs were derived from previous analyses and converted to 2011 I$ as described above. Cancer care costs in India were based on primary data [[14](#_ENREF_14)], while costs from Uganda were based on primary data from Kenya, as we have previously described [[14](#_ENREF_14),[15](#_ENREF_15)]. Cancer care costs from Nicaragua were based on primary data from the cost of treating cancer in El Salvador (excluding staging costs) [[16](#_ENREF_16)]. To adjust cancer costs from El Salvador to the setting of Nicaragua, we assumed direct medical costs were reduced by the ratio of WHO-CHOICE inpatient bed-day costs at a teaching hospital (for cancer center procedures) or WHO-CHOICE outpatient procedures at a secondary-level hospital (for regular follow-up care after cancer treatment) in Nicaragua relative to El Salvador; women’s time costs were reduced by the ratio of wages (in 2011 I$) in Nicaragua relative to El Salvador, and transportation and incidental costs were reduced by the ratio of GNI per capita (in 2011 I$). Costs are reported in **Table 1** of the main manuscript.

**Women’s Time and Transportation Costs**

We derived women’s time costs from the United Nations Development Programme Human Development Indicator, “Estimated GNI per capita, female”, which was derived from the ratio of female to male wage, female and male shares of economically active population, and gross national income (GNI) and reported in constant 2011 I$ [[17](#_ENREF_17)]. We assumed this represented annual income for working 40 hours per week, 50 weeks per year to estimate an average hourly wage (**Table 1**).

Estimates for time spent traveling, waiting, and receiving care was dependent upon the facility level where care was assumed to take place (**Table A.7**). Women’s time estimates for round-trip transportation and waiting were obtained from prior studies in El Salvador (for Nicaragua) [[16](#_ENREF_16)], India, and Kenya (for Uganda) (**Table A.8**) [[14](#_ENREF_14),[18](#_ENREF_18)]. Estimates of women’s time spent receiving a procedure were based on site-specific data from the START-UP demonstration projects, with staff time spent on the procedure (excluding preparation and registration time, which we assumed were built into patient waiting time) used as a proxy for women’s procedure time. Round-trip transportation costs to each health facility level were obtained from previous analyses [[14](#_ENREF_14),[15](#_ENREF_15),[18](#_ENREF_18)] and converted to 2011 I$; these are reported in **Table 1** of the main manuscript.

**PROTOCOLS FOR TREATMENT OF PRECANCER**

Women who screened positive with HPV DNA testing who were ineligible for cryotherapy were assumed to be referred to a secondary facility for colposcopy and subsequent treatment. Treatment protocols were based on information from in-country clinicians familiar with standard of care and availability of and preferences for treatment options. In Hyderabad, we assumed that, upon a histologic diagnosis of CIN1, CIN2, or CIN3, women received cryotherapy at a secondary facility. In Nicaragua, we assumed that a histologic diagnosis of CIN1 was followed by cryotherapy and CIN2/3 was followed by LEEP at a secondary facility. In Uganda, we assumed that, upon a histologic diagnosis of CIN1, women received cryotherapy at a secondary facility; a histologic diagnosis of CIN2/3 was followed by cryotherapy for approximately 80% of women, and LEEP for approximately 20% of women, and treatment occurred at a secondary facility.

Loss-to-follow-up rates impact cost accrual in the microsimulation model, and we have the flexibility to input differential loss-to-follow-up for each visit (i.e., results, cryotherapy (if delayed), diagnostic confirmation, and treatment following diagnostic confirmation). In the base case, we assumed 10% of women would be lost to follow-up if subject to delayed cryotherapy in a screen-and-treat strategy (i.e., VIA or HPV testing). We assumed visits for screening results, diagnostic confirmation, and treatment following diagnostic confirmation were each associated with 15% loss-to-follow-up.

Following treatment of precancerous lesions with either cryotherapy or LEEP, we assumed the setting-specific follow-up protocols as used in the START-UP demonstration studies (**Table A.9**). We included direct medical costs of each procedure, as well as women’s time and transportation costs (as shown in **Table 1** of the main manuscript). While women in the START-UP studies could be seen prior to scheduled follow-up visits as necessary, we did not have data on these unscheduled visits. Treatment complications in each site were very rare, so we did not consider these costs in the base case analysis.

**EQUATION FOR CALCULATION OF INCREMENTAL NET MONETARY BENEFIT (INMB)**

$$\Delta Life expectancy \times WTP- \Delta Cost$$

where

$\Delta Life expectancy=({Life expectancy}_{improved}$ $- {Life expectancy}_{current}$)

$\Delta Cost=({Cost}_{improved}-{Cost}_{current}$)

and WTP = willingness-to-pay threshold

In this analysis, 1-visit screening was considered the “improved” strategy, whereas 2-visit screening was considered the “current”, or status quo, strategy.

**SUPPLEMENTAL RESULTS**

Results assuming a WTP threshold of three times GDP per capita are displayed in **Table A.10.**

**Table A.1. Age-specific prevalence of high-risk HPV, India [**[**19**](#_ENREF_19)**].**^a^

| **Age group** | **Number of women** | **Number of women with high-risk HPV** | **Prevalence (95% CI)** |
| --- | --- | --- | --- |
| 30 – 34 years | 1,949 | 214 | 0.11 (0.10, 0.13) |
| 35 – 39 years | 1,158 | 99 | 0.09 (0.07, 0.10) |
| 40 – 44 years | 708 | 76 | 0.11 (0.09, 0.13) |
| 45 – 49 years | 687 | 85 | 0.12 (0.10, 0.15) |

^a^ HPV positivity was based on a cut-off of 0.5 relative light units.

**Table A.2. Age-specific prevalence of high-risk HPV, Nicaragua [**[**19**](#_ENREF_19)**].**^a^

| **Age group** | **Number of women** | **Number of women with high-risk HPV** | **Prevalence (95% CI)** |
| --- | --- | --- | --- |
| 30 – 34 years | 1,693 | 310 | 0.18 (0.17, 0.20) |
| 35 – 39 years | 1,141 | 184 | 0.16 (0.14, 0.18) |
| 40 – 44 years | 933 | 125 | 0.13 (0.11, 0.16) |
| 45 – 49 years | 878 | 121 | 0.14 (0.12, 0.16) |

^a^ HPV positivity was based on a cut-off of 0.5 relative light units.

**Table A.3. Age-specific prevalence of high-risk HPV, Uganda [**[**19**](#_ENREF_19)**].**^a^

| **Age group** | **Number of women** | **Number of women with high-risk HPV** | **Prevalence (95% CI)** |
| --- | --- | --- | --- |
| 25 – 34 years | 1,367 | 426 | 0.31 (0.28, 0.34) |
| 35 – 44 years | 1,131 | 284 | 0.25 (0.23, 0.28) |
| 45 – 54 years | 558 | 127 | 0.22 (0.19, 0.26) |
| 55 – 60 years | 90 | 28 | 0.31 (0.22, 0.42) |

^a^ HPV positivity was based on a cut-off of 0.5 relative light units. We did not include HPV prevalence in our scoring algorithm for Uganda, although we did consider visual fit to HPV prevalence.

**Table A.4. Age-specific cervical cancer incidence, India (Nagpur registry, 1998-2002)[**[**11**](#_ENREF_11)**].**^a^

| **Age group** | **Cases** | **Rate per 100,000 women (95% CI)** |
| --- | --- | --- |
| 20 – 24 years | 9 | 1.8 (0.6, 2.9) |
| 25 – 29 years | 11 | 2.3 (0.9, 3.6) |
| 30 – 34 years | 43 | 10.6 (7.4, 13.7) |
| 35 – 39 years | 62 | 16.9 (12.7, 21.1) |
| 40 – 44 years | 90 | 32.4 (25.7, 39.1) |
| 45 – 49 years | 107 | 46.2 (37.5, 55.0) |
| 50 – 54 years | 105 | 58.9 (47.6, 70.1) |
| 55 – 59 years | 70 | 52.4 (40.1, 64.7) |
| 60 – 64 years | 104 | 75.0 (60.6, 89.4) |
| 65 – 69 years | 71 | 62.5 (47.9, 77.0) |
| 70 – 74 years | 44 | 57.6 (40.6, 74.6) |
| ≥75 years | 9 | 26.8 (9.3, 44.2) |

^a^ Although our scoring algorithm included cancer incidence in women aged 30 to 49 years, we considered visual fit to all age groups.

**Table A.5. Age-specific cervical cancer incidence, Nicaragua (GLOBOCAN 2012) [**[**12**](#_ENREF_12)**].**

| **Age group** | **Cases** | **Rate per 100,000 women (95% CI)** |
| --- | --- | --- |
| 40 – 44 years | 123 | 78.7 (64.8, 92.6) |
| 45 – 49 years | 112 | 85.4 (69.6, 101.2) |
| 50 – 54 years | 102 | 88.4 (71.2, 105.6) |
| 55 – 59 years | 85 | 88.1 (69.4, 106.8) |
| 60 – 64 years | 51 | 84.0 (61.0, 107.1) |
| 65 – 69 years | 37 | 80.8 (54.8, 106.8) |
| 70 – 74 years | 30 | 74.6 (47.9, 101.3) |
| ≥75 years | 45 | 70.3 (49.8, 90.8) |

**Table A.6. Age-specific cervical cancer incidence, Uganda (Kyadondo registry, 2003-2007)[**[**11**](#_ENREF_11)**].**^a^

| **Age group** | **Cases** | **Rate per 100,000 women (95% CI)** |
| --- | --- | --- |
| 25 – 29 years | 42 | 7.6 (5.3, 9.9) |
| 30 – 34 years | 84 | 26.5 (20.8, 32.2) |
| 35 – 39 years | 111 | 53.7 (43.7, 63.7) |
| 40 – 44 years | 138 | 99.7 (83.1, 116.3) |
| 45 – 49 years | 105 | 121.7 (98.4, 145.0) |
| 50 – 54 years | 108 | 181.3 (147.1, 215.5) |
| 55 – 59 years | 59 | 163.2 (121.6, 204.8) |
| 60 – 64 years | 68 | 199.7 (152.2, 247.2) |
| 65 – 69 years | 33 | 145.8 (96.1, 195.6) |
| 70 – 74 years | 35 | 175.0 (117.0, 233.0) |

^a^ Although our scoring algorithm included cancer incidence in aged 40 years and above, we considered visual fit to all age groups.

**Table A.7. Location of Service Delivery for Screening, Diagnosis, and Treatment of Precancerous Lesions and Cancer.**^a^

| **Procedure** | | **Location of services** |
| --- | --- | --- |
| HPV DNA test | | Primary facility |
| Cytology test | | Primary facility |
| VIA test | | Primary facility |
| Colposcopy/biopsy | | Secondary facility |
| Cryotherapy | | Primary facility (for women eligible for screen-and-treat cryotherapy)  Secondary facility (for women ineligible for screen-and-treat cryotherapy |
| LEEP | | Secondary facility |
| Follow-up visits (after cryotherapy or LEEP) | | Primary facility (for examinations and Pap)  Secondary facility (if colposcopy is necessary) |
| Cancer treatment | | Tertiary facility |
| ^a^ | HPV: human papillomavirus; LEEP: loop electrosurgical excision procedure; VIA: visual inspection with acetic acid. | |

**Table A.8. Women’s Time Spent Receiving Care.**^a^

| **Procedure** | | **Time Spent Receiving Care (Minutes)** | | | |
| --- | --- | --- | --- | --- | --- |
|  | |  | **Hyderabad[**[**14**](#_ENREF_14)**,**[**19**](#_ENREF_19)**]** | **Nicaragua[**[**18**](#_ENREF_18)**,**[**19**](#_ENREF_19)**]** | **Uganda[**[**14**](#_ENREF_14)**,**[**19**](#_ENREF_19)**]** |
| **Screening** | | | | | |
| Wait time  Procedure time  Transport time (round-trip) | | | 60 | 15 | 90 |
|  |  |  | 15 | 20 | 15 |
|  |  |  | 60 | 90 | 220 |
| Receiving results (negative)^b^  5 2 10 | | | | | |
| Receiving results (positive) 15 5 15 | | | | | |
| **Diagnosis** | | | | | |
| Wait time | | | 120 | 150 | 180 |
| Procedure time  Transport time (round-trip) | | | 42 | 37 | 35 |
|  |  |  | 240 | 90 | 340 |
|  | | | | | |
| **Treatment of Precancer: Screen-and-Treat Cryotherapy** ^c^ | | | | | |
| Wait time  Procedure time  Transport time (round-trip) | | | 60 | 150 | 90 |
|  |  |  | 30 | 35 | 30 |
|  |  |  | 60 | 90 | 220 |
|  | | | | | |
| **Treatment of Precancer: LEEP** | | | | | |
| Wait time  Procedure time  Transport time (round-trip) | | | NA | 150 | 180 |
|  |  |  | NA | 25 | 25 |
|  |  |  | NA | 90 | 340 |
| ^a^ | I$: international dollars. LEEP: loop electrosurgical excision procedure. | | | | |
| ^b^ | Applicable to careHPV and Pap screening. | | | | |
| ^c^ | We assumed most eligible women received cryotherapy in the same visit they received screening results (i.e., initial screening visit with VIA; second visit for HPV testing). Thus, additional transportation time was only accrued for women who delayed cryotherapy. Screen-and-treat cryotherapy was assumed to take place at a primary facility. For women who received cryotherapy following diagnostic confirmation of CIN, wait time and transport time were the same as for LEEP, as cryotherapy was assumed to take place at a secondary facility. | | | | |

**Table A.9. Follow-up Protocols after Treatment of Precancerous Lesions.**^a^

| **Treatment** | |  | **Hyderabad** | **Nicaragua** | **Uganda** |
| --- | --- | --- | --- | --- | --- |
| **Cryotherapy** | | | | | |
|  | |  |  |  | 6 week exam |
|  | |  | 1 year Cytology/Colposcopy | 1 year Cytology | 1 year Cytology/Colposcopy^b^ |
|  | | | | | |
| **LEEP** | | | | | |
|  | |  | NA | 1 year Cytology | 6 week exam |
|  | |  |  |  | 1 year Cytology  1 year Colposcopy, as needed^b^ |
| a | LEEP: loop electrosurgical excision procedure. Follow-up protocols were based on the START-UP demonstration study in each setting. We included direct medical costs and women’s time and transportation costs for each procedure. A 6 week visual exam was associated with the same costs as VIA at the primary facility. Cytology was assumed to take place at a primary facility, while colposcopy was assumed to take place at a secondary facility. | | | | |
| b | Colposcopy at 1 year was performed as needed in Uganda. Approximately 15% of women who received treatment required a colposcopy and biopsy at 1 year for suspected recurrence. | | | | |

**Table A.10. Health outcomes, costs, and incremental net monetary benefits of reducing the number of visits for cervical cancer screening and treatment of precancer treatment, considering a willingness-to-pay threshold of three times GDP per capita.**^a^

|  | **India (GDP per capita: I$5,450)** | | | | **Nicaragua (GDP per capita: I$4,690)** | | | | **Uganda (GDP per capita: I$1,690)** | | | |
| --- | --- | --- | --- | --- | --- | --- | --- | --- | --- | --- | --- | --- |
| **Scenario**^b^ | **Cancer incidence reduction, %**^c^ | **Lifetime cost (2011 I$)**^d^ | **Life expectancy**^e^ | **INMB**  **(3x per capita GDP threshold)**^f^ | **Cancer incidence reduction, %**^c^ | **Lifetime cost (2011 I$)**^d^ | **Life expectancy**^e^ | **INMB**  **(3x per capita GDP threshold)**^f^ | **Cancer incidence reduction, %**^c^ | **Lifetime cost (2011 I$)**^d^ | **Life expectancy**^e^ | **INMB**  **(3x per capita GDP threshold)**^f^ |
| No screening | -- | 8.87  (7.05-12.54) | 27.78539  (27.76486-27.79362) | -- | -- | 42.67 | 28.58210 | -- | -- | 12.42  (10.96-14.16) | 25.20221  (25.17432-25.22855) | -- |
| **LTFU: 10%** | | | | | | | | | | | | |
| 2-visit | 62.0  (48.8-76.6) | 29.68  (27.25-32.02) | 27.82880  (27.82371-27.83431) | -- | 66.0  (58.9-73.6) | 53.03  (48.86-56.89) | 28.73581  (28.72337-28.74977) | -- | 67.4  (62.3-73.1) | 45.66  (43.01-47.78) | 25.33856  (25.33166-25.34855) | -- |
| 1-visit | 65.0  (51.8-79.4) | 27.32  (24.81-29.72) | 27.83081  (27.82611-27.83594) | 35  (28-44) | 68.8  (61.7-76.3) | 48.69  (44.60-52.56) | 28.74246  (28.73123-28.75554) | 98  (78-116) | 70.1  (65.0-75.9) | 37.75  (34.87-40.12) | 25.34406  (25.33770-25.35278) | 36  (29-40) |
| **LTFU: 20%** | | | | | | | | | | | | |
| 2-visit | 58.1  (45.1-72.8) | 28.92  (26.55-31.22) | 27.82567  (27.82006-27.83160) | -- | 61.9  (54.8-69.5) | 53.51  (49.16-57.58) | 28.72533  (28.71125-28.74107) | -- | 63.2  (58.1-68.8) | 43.91  (41.45-45.79) | 25.32940  (25.32187-25.34090) | -- |
| 1-visit | 64.7  (51.4-79.1) | 27.31  (24.79-29.72) | 27.83013  (27.82534-27.83545) | 75  (61-92) | 68.2  (61.1-75.8) | 48.82  (44.72-52.74) | 28.74042  (28.72857-28.75389) | 217  (176-255) | 69.6  (64.6-75.4) | 37.70  (34.81-40.07) | 25.34217  (25.33572-25.35127) | 71  (59-80) |
| **LTFU: 30%** | | | | | | | | | | | | |
| 2-visit | 53.5  (40.9-68.0) | 28.21  (25.91-30.45) | 27.82200  (27.81527-27.82855) | -- | 57.2  (50.2-64.5) | 54.25  (49.76-58.61) | 28.71253  (28.69672-28.73052) | -- | 58.4  (53.6-64.0) | 42.21  (39.94-43.82) | 25.31800  (25.30857-25.33155) | -- |
| 1-visit | 64.3  (50.9-78.9) | 27.31  (24.77-29.73) | 27.82940  (27.82447-27.83494) | 122  (103-158) | 67.8  (60.7-75.4) | 48.93  (44.80-52.87) | 28.73814  (28.72585-28.75192) | 366  (302-432) | 69.2  (64.2-75.1) | 37.65  (34.76-40.02) | 25.34011  (25.33346-25.34958) | 117  (96-133) |
| **LTFU: 40%** | | | | | | | | | | | | |
| 2-visit | 48.4  (36.2-62.9) | 27.54  (25.26-29.73) | 27.78180  (27.81012-27.82500) | -- | 51.8  (45.3-59.0) | 55.24  (50.64-59.90) | 28.69851  (28.68045-28.71844) | -- | 52.8  (48.3-58.3) | 40.61  (38.52-41.95) | 25.30545  (25.29383-25.32110) | -- |
| 1-visit | 64.0  (50.4-78.9) | 27.30  (24.74-29.73) | 27.82872  (27.82352-27.83453) | 176  (151-230) | 67.5  (60.5-75.2) | 48.99  (44.80-52.92) | 28.73577  (28.72275-28.75003) | 531  (448-624) | 68.9  (63.8-74.8) | 37.60  (34.71-39.97) | 25.33789  (25.33107-25.34820) | 167  (141-193) |
| **LTFU: 50%** | | | | | | | | | | | | |
| 2-visit | 42.6  (31.3-56.2) | 26.92  (24.71-29.22) | 27.81361  (27.80433-27.82065) | -- | 45.6  (39.5-52.2) | 56.62  (51.93-61.68) | 28.68319 (28.66223-28.70486) | -- | 46.4  (42.4-51.5) | 39.08  (27.19-40.42) | 25.29166  (25.27809-25.30957) | -- |
| 1-visit | 63.9  (50.2-78.9) | 27.29  (24.70-29.70) | 27.82818  (27.82289-27.83407) | 238  (208-314) | 67.2  (60.2-75.0) | 49.01  (44.81-52.95) | 28.73386 (28.72017-28.74855) | 721  (621-850) | 68.6  (63.5-74.5) | 37.54  (34.66-39.91) | 25.33606  (25.32892-25.34684) | 227  (191-260) |
| **LTFU: 60%** | | | | | | | | | | | | |
| 2-visit | 35.7  (25.7-47.8) | 26.39  (24.31-28.90) | 27.80868  (27.79781-27.81552) | -- | 38.3  (32.9-44.2) | 58.38  (53.62-63.84) | 28.66565  (28.65149-28.68919) | -- | 39.1  (35.4-43.6) | 37.66  (35.86-39.03) | 25.27631  (25.26027-25.29619) | -- |
| 1-visit | 63.7  (49.9-78.7) | 27.28  (24.69-29.71) | 27.82763  (27.82222-27.83379) | 309  (267-412) | 67.0  (60.0-74.9) | 49.04  (44.83-52.96) | 28.73182  (28.71765-28.74704) | 940  (820-1,110) | 68.4  (63.2-74.3) | 37.49  (34.60-39.87) | 25.33429  (25.32690-25.34550) | 294  (251-339) |
| **LTFU: 70%** | | | | | | | | | | | | |
| 2-visit | 28.2  (20.3-37.9) | 25.89  (23.96-28.62) | 27.80362  (27.79067-27.81054) | -- | 30.2  (26.0-35.2) | 60.44  (55.49-66.41) | 28.64712  (28.62014-28.67254) | -- | 30.9  (27.9-34.7) | 36.33  (34.63-37.76) | 25.26014  (25.32487-25.34428) | -- |
| 1-visit | 63.5  (49.6-78.7) | 27.27  (24.68-29.69 | 27.82722  (27.82182-27.83347) | 384  (323-522) | 66.9  (59.9-74.8) | 49.04  (44.82-52.97) | 28.72980  (28.71518-28.74542) | 1,175  (1,029-1377) | 68.3  (63.1-74.2) | 37.44  (34.55-39.82) | 25.33251  (25.32487-25.34428) | 366  (316-422) |

^a^ Average values represent the outcomes using 50 calibrated parameter sets for each country; parentheses indicate the minimum and maximum values across 50 calibrated parameter sets. GDP: gross domestic product; I$: international dollars; INMB: incremental net monetary benefit; LTFU: loss to follow-up.

^b^ Scenarios are listed in order of increasing health benefit, and include the following for women aged 30, 35, and 40 years: 2-visit screen-and-treat, 1-visit screen-and-treat, and home-based screening with instantaneous results followed by a clinic visit for screen-positive women only. Under all scenarios, screening coverage was 100%, with LTFU for each clinical encounter. For the 2-visit scenario, LTFU applied to the results/treatment visit; for all scenarios, LTFU applied to diagnostic testing with colposcopy and treatment of colposcopically confirmed CIN1+ for women who were not eligible for immediate cryotherapy at a primary facility.

^c^ Cancer incidence reduction reflects the percent reduction in lifetime risk of cancer incidence compared to no screening.

^d^ Total discounted lifetime cost per woman.

^e^ Total discounted life expectancy.

^f^ The INMB for each scenario is calculated against 2-visit screen-and-treat, at the specified level of LTFU. The INMB for 1-visit screen-and-treat and home-based screening provide a measure of how much economic investment could be made per woman to achieve a reduction in visits (relative to the 2-visit screen-and-treat scenario) without exceeding a country’s willingness to pay (WTP), at each level of LTFU. We considered each country’s WTP to be equivalent to three times GDP per capita.

**Figure A1.** Selected model output from the top 50 input parameter sets compared with empirical data (i.e., calibration targets) on age-specific prevalence of high-risk HPV in India (Hyderabad), based on a relative light unit cut-off value of 0.5 in the START-UP studies [[19](#_ENREF_19)]. Bold lines represent the 95% confidence intervals around the empirical data, and gray circles represent model output from each of the top 50 input parameter sets.

**Figure A2.** Selected model output from the top 50 input parameter sets compared with empirical data (i.e., calibration targets) on age-specific prevalence of high-risk HPV in Nicaragua, based on a relative light unit cut-off value of 0.5 in the START-UP studies [[19](#_ENREF_19)]. Bold lines represent the 95% confidence intervals around the empirical data, and gray circles represent model output from each of the top 50 input parameter sets.

**Figure A3.** Selected model output from the top 50 input parameter sets compared with empirical data (i.e., calibration targets) on age-specific prevalence of high-risk HPV in Uganda, based on a relative light unit cut-off value of 0.5 in the START-UP studies [[19](#_ENREF_19)]. Bold lines represent the 95% confidence intervals around the empirical data, and gray circles represent model output from each of the top 50 input parameter sets.

**Figure A4.** Selected model output from the top 50 input parameter sets compared with empirical data (i.e., calibration targets) on age-specific cancer incidence in India (Nagpur registry, 1998-2002)[[11](#_ENREF_11)]. Bold lines represent the 95% confidence intervals around the empirical data, and gray circles represent model output from each of the top 50 input parameter sets.

**Figure A5.** Selected model output from the top 50 input parameter sets compared with empirical data (i.e., calibration targets) on age-specific cancer incidence in Nicaragua (GLOBOCAN 2012). Bold lines represent the 95% confidence intervals around the empirical data, and gray circles represent model output from each of the top 50 input parameter sets.

**Figure A6.** Selected model output from the top 50 input parameter sets compared with empirical data (i.e., calibration targets) on age-specific cancer incidence in Uganda (Kyadondo registry, 2003-2007)[[11](#_ENREF_11)]. Bold lines represent the 95% confidence intervals around the empirical data, and gray circles represent model output from each of the top 50 input parameter sets.

**References**

1. Campos NG, Burger EA, Sy S, Sharma M, Schiffman M, et al. (2014) An updated natural history model of cervical cancer: derivation of model parameters. Am J Epidemiol 180: 545-555.

2. Campos NG, Tsu, V., Jeronimo, J., Mvundura, M., Lee, K., Kim, J.J. (2015) When and how often to screen for cervical cancer in three low- and middle-income countries: A cost-effectiveness analysis. Papillomavirus Research.

3. Munoz N, Mendez F, Posso H, Molano M, van den Brule AJ, et al. (2004) Incidence, duration, and determinants of cervical human papillomavirus infection in a cohort of Colombian women with normal cytological results. J Infect Dis 190: 2077-2087.

4. Herrero R, Hildesheim A, Rodriguez AC, Wacholder S, Bratti C, et al. (2008) Rationale and design of a community-based double-blind randomized clinical trial of an HPV 16 and 18 vaccine in Guanacaste, Costa Rica. Vaccine 26: 4795-4808.

5. McCredie MR, Sharples KJ, Paul C, Baranyai J, Medley G, et al. (2008) Natural history of cervical neoplasia and risk of invasive cancer in women with cervical intraepithelial neoplasia 3: a retrospective cohort study. Lancet Oncol 9: 425-434.

6. Meyskens FL, Jr., Surwit E, Moon TE, Childers JM, Davis JR, et al. (1994) Enhancement of regression of cervical intraepithelial neoplasia II (moderate dysplasia) with topically applied all-trans-retinoic acid: a randomized trial. J Natl Cancer Inst 86: 539-543.

7. Keefe KA, Schell MJ, Brewer C, McHale M, Brewster W, et al. (2001) A randomized, double blind, Phase III trial using oral beta-carotene supplementation for women with high-grade cervical intraepithelial neoplasia. Cancer Epidemiol Biomarkers Prev 10: 1029-1035.

8. Castle PE, Schiffman M, Wheeler CM, Solomon D (2009) Evidence for frequent regression of cervical intraepithelial neoplasia-grade 2. Obstet Gynecol 113: 18-25.

9. Wang SM, Colombara D, Shi JF, Zhao FH, Li J, et al. (2013) Six-year regression and progression of cervical lesions of different human papillomavirus viral loads in varied histological diagnoses. Int J Gynecol Cancer 23: 716-723.

10. Moscicki AB, Ma Y, Wibbelsman C, Darragh TM, Powers A, et al. (2010) Rate of and risks for regression of cervical intraepithelial neoplasia 2 in adolescents and young women. Obstet Gynecol 116: 1373-1380.

11. Forman D, Bray, F., Brewster, D.H., Gombe Mbalawa, C., Kohler, B., Piñeros, M., Steliarova-Foucher, E., Swaminathan, R., Ferlay, J. (eds) (2013) Cancer Incidence in Five Continents, Vol. X. Lyon, France: IARC.

12. Ferlay J, Soerjomataram I, Ervik M, Dikshit R, Eser S, et al. (2013) GLOBOCAN 2012 v1.0, Cancer Incidence and Mortality Worldwide: IARC CancerBase No. 11 [Internet]. Lyon, France: International Agency for Research on Cancer.

13. (2016) World Development Indicators. World Bank.

14. Goldie SJ, Gaffikin L, Goldhaber-Fiebert JD, Gordillo-Tobar A, Levin C, et al. (2005) Cost-effectiveness of cervical-cancer screening in five developing countries. N Engl J Med 353: 2158-2168.

15. Campos NG, Kim JJ, Castle PE, Ortendahl JD, O'Shea M, et al. (2012) Health and economic impact of HPV 16/18 vaccination and cervical cancer screening in Eastern Africa. Int J Cancer 130: 2672-2684.

16. Campos NG, Maza M, Alfaro K, Gage JC, Castle PE, et al. (2015) The comparative and cost-effectiveness of HPV-based cervical cancer screening algorithms in El Salvador. Int J Cancer.

17. Programme UND (2014) International Human Development Indicators.

18. Campos NGM, M.; Alfaro, K.; Gage, J.C.; Castle, P.E.; Felix, J.; Cremer, M.L.; Kim, J.J. The Comparative and Cost-Effectiveness of HPV-Based Cervical Cancer Screening in El Salvador.

19. Jeronimo J, Bansil P, Lim J, Peck R, Paul P, et al. (2014) A multicountry evaluation of careHPV testing, visual inspection with acetic acid, and papanicolaou testing for the detection of cervical cancer. Int J Gynecol Cancer 24: 576-585.
